# The increased trend in mothers’ hospital admissions for psychiatric disorders in the first year after birth between 2001 and 2010 in New South Wales, Australia

**DOI:** 10.1186/1472-6874-14-119

**Published:** 2014-09-29

**Authors:** Fenglian Xu, Elizabeth A Sullivan, Zhuoyang Li, Lucy Burns, Marie-Paule Austin, Tim Slade

**Affiliations:** National Drug and Alcohol Research Centre, University of New South Wales, Sydney, NSW 2031 Australia; Faculty of Health, University of Technology Sydney, Sydney, NSW 2007 Australia; Perinatal and Women’s Mental Health Unit, St John of God Health Care and School of Psychiatry, University of New South Wales, Sydney, NSW 2052 Australia

**Keywords:** Mental health, Increasing trend, Postpartum, Data linkage

## Abstract

**Background:**

The burden of mental and behavioural disorders in Australia has increased significantly over the last decade. The aim of the current study is to describe the hospital admission rates for mental illness over a 10-year period for primiparous mothers in the first year after birth.

**Methods:**

This is an Australian population-based descriptive study with linked data from the New South Wales Midwives Data Collection and Admitted Patients Data Collection. The study population included primiparous mothers who gave birth between 1 January 2001 and 31 December 2010. All hospital admissions with a mental health diagnosis in the first year after birth were recorded.

**Results:**

There were 6,140 mothers (1.67%) admitted to hospital with a principal diagnosis of mental health in the first year after birth between 2001 and 2010 in New South Wales (7,884 admissions, 2.15%). The hospital admission rates increased significantly over time, particularly from 2005. The increase in hospital admissions was mainly attributed to the diagnoses of unipolar depression, adjustment disorders and anxiety disorders.

**Conclusions:**

This study shows that hospital admissions for mothers with a mental health diagnosis after birth in New South Wales has significantly increased in the last decade. Possible reasons for this change need to be studied further.

## Background

The burden of mental and behavioural disorders (MBD) in Australia has increased significantly over the last decade, and this is reflected in a rise in the use of health services. The Australian Institute of Health and Welfare (AIHW) reported that the number of hospital admissions for MBD increased more than 40% between 1998–1999 and 2009–2010, with the greatest increase due to stress, somatoform disorders and neurotic (F40 - F48), mood (affective) disorders (F30 - F39) and psychoactive substance use (F10 - F19) [1]. In 2010–2011, almost $6.9 billion was spent on mental health-related service delivery in Australia, an increase of 5.7% per Australian compared with 2006–2007 [2].

Postpartum women are a special population. Literature based on population data reported an increase in hospital admissions for MBD in the first year after birth [3–5]. It has been estimated that postpartum depression can affect up to 15% of women [3, 6]. The morbidity and costs associated with maternal MBD in this period is substantial for mothers and their families [7]. A systematic review showed that as many as 6.5% of mothers have a new episode of major depression during the first three months after delivery [8]. A Danish population-based cohort study showed that the rate of first-time hospital admission for mental disorders was 0.6% in the first month after birth [5]. However, there is little literature on trends in hospital admissions for postpartum mental illness over time in Australia. There have been a few state-wide initiatives, including education initiatives, which have increased awareness among medical providers and the public. The impact of these initiatives needs to be assessed.

To address this gap in research, we used population-based linked data to answer two specific questions: (1) What are hospital admission rates for mental disorders over time? (2) What are the risk factors for hospital admissions for mental health one year following birth?

## Methods

### Study population

The study included all primiparous women who gave birth in New South Wales (NSW) between 1 January 2001 and 31 December 2010, and who did not give birth for the second time within one year (365 days). For plural births, the first birth record was used for the analysis.

### Study design

This was a population-based descriptive study with linked data from the NSW Midwives Data Collection (MDC) and the NSW Admitted Patients Data Collection (APDC). MDC birth records from 1 January 2001 to 31 December 2010 were linked with APDC records between 1 January 2001 and 31 December 2011. All hospital admissions for mental health disorders in the first year after birth were recorded.

The MDC is a data collection of all births of at least 20 weeks gestation or at least 400 grams birthweight in NSW. It covers all births in public and private hospitals as well as home births. The APDC is a routinely collected census of all hospital separations. It includes all patient hospitalisations in NSW public and private hospitals including psychiatric hospitals and day procedures. Since 1999, diagnoses for hospital admissions have been coded according to the 10th revision of the International Statistical Classification of Diseases and Related Health Problems, Australian Modification (ICD-10-AM) [9].

### Definitions of diagnosis in APDC

- Principal diagnosis is the diagnosis which was chiefly responsible for hospital admissions [10].
- Non-principal diagnoses include stay and other diagnoses. In NSW APDC data, the diagnoses in each hospital admission record include one principal diagnosis, one stay diagnosis and 53 other diagnoses. ‘Stay diagnosis’ refers to the diagnosis that most influenced the length of stay in hospital [11]. ‘Other diagnosis’ is an additional diagnosis and refers to a condition or a complaint either coexisting with the principal and stay diagnoses or arising during the hospitalisation [11].
- All diagnoses include principal diagnosis and non-principal diagnoses.
- Psychiatric disorders postpartum refer to the diagnoses of F00–99 excluding substance use disorders (F10–19) in the first year after birth (including the day of birth).

Mothers with mental health diagnoses were identified using ICD-10-AM diagnosis codes: (1) depressive episode (F32); (2) anxiety disorders (F41); (3) adjustment disorders (F43); (4) puerperal mental disorders (F53); all diagnoses include the diagnoses of F00–99 and exclude substance use disorders (F10–19).

Data linkage for this study was performed by the Centre for Health Record Linkage (CHeReL) at the NSW Ministry of Health using ChoiceMaker software [12]. This software is a comprehensive and extensible suite of Java-based software that finds matches between people’s electronic records and allows users to make use of stacked data [10]. Based on the 1000 randomly selected sample of records, the false positive rate of the data linkage was 0.3%.

### Statistical analysis

Descriptive statistics, percentage and 95% confidence intervals (CIs) were used to show the hospital admission rate and differences over years. If the 95% CI of two rates does not overlap, the rates are significantly different (p < 0.05). However, the converse, that is two rates are not significantly different if their CIs are overlapped, is not necessarily true. The chi-square test was used to determine the overall difference in the rates. Binary logistic regression was used to calculate the odds ratio (OR) and 95% CI. This OR represents the odds of hospital admissions for mental illness over years. For OR interpretation, the reference year is 2001, the risk for hospital admissions for mental illness (ORs) between 2002 and 2010 are compared with 2001. The analyses were conducted using IBM SPSS (Statistical Package for the Social Sciences) Statistics 20 [13].

### Ethics approval

This study was approved by the NSW Population & Health Services Research Ethics Committee and the Human Research Ethics Committee of the University of New South Wales, Australia.

## Results

**Figure 1:**
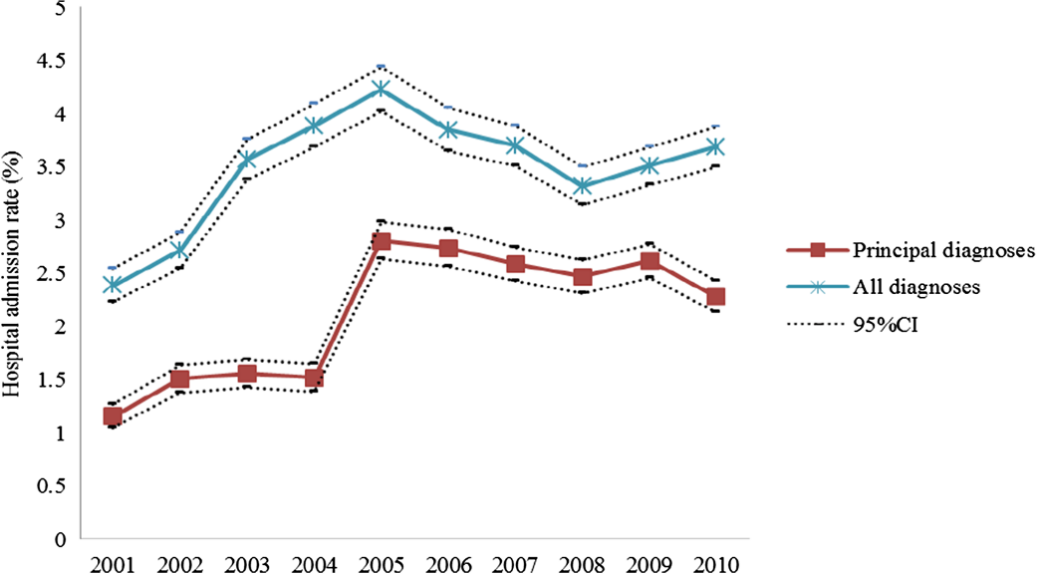
**Hospital admissions for psychiatric disorders in primiparous mothers, 2001–2010, New South Wales, Australia.** CI: confidence interval.

There were 367,155 primiparous mothers who gave birth between 2001 and 2010 in NSW. Among them, 6,140 mothers (1.67%) were admitted to hospital for a psychiatric disorder as the principal diagnosis (7,884 admissions, 2.15%), and 10,160 mothers (2.77%) were admitted to hospital with a psychiatric disorder for either a principal or non-principal diagnosis (12,836 admissions, 3.50%) in the first year after birth (Table 1; Figure 1).

**Table 1:** **Hospital admissions for psychiatric disorders in primiparous mothers, 2001–2010, New South Wales, Australia**

Year	Primiparous mothers (n)	Principal diagnosis				All diagnoses			
		Mothers	Rate (%)	95% CI		Mothers	Rate (%)	95% CI	
2001	34,816	273	0.78	0.69	0.87	615	1.77	1.63	1.91
2002	34,463	430	1.25	1.13	1.37	760	2.21	2.05	2.37
2003	35,091	359	1.02	0.91	1.13	952	2.71	2.54	2.88
2004	34,877	436	1.25	1.13	1.37	1,145	3.28	3.09	3.47
2005	36,157	860	2.38	2.22	2.54	1,299	3.59	3.40	3.78
2006	36,509	779	2.13	1.98	2.28	1,088	2.98	2.81	3.15
2007	38,379	761	1.98	1.84	2.12	1,064	2.77	2.61	2.93
2008	38,330	771	2.01	1.87	2.15	1,018	2.66	2.50	2.82
2009	38,960	752	1.93	1.79	2.07	1,030	2.64	2.48	2.80
2010	39,573	719	1.82	1.69	1.95	1,189	3.00	2.83	3.17
Total	367,155	6140	1.67	1.63	1.71	10,160	2.77	2.72	2.82
		Hospital admissions^#^				Hospital admissions^#^			
2001	34,816	404	1.16	1.05	1.27	832	2.39	2.23	2.55
2002	34,463	521	1.51	1.38	1.64	938	2.72	2.55	2.89
2003	35,091	548	1.56	1.43	1.69	1,251	3.57	3.38	3.76
2004	34,877	530	1.52	1.39	1.65	1,357	3.89	3.69	4.09
2005	36,157	1015	2.81	2.64	2.98	1,530	4.23	4.02	4.44
2006	36,509	1001	2.74	2.57	2.91	1,407	3.85	3.65	4.05
2007	38,379	994	2.59	2.43	2.75	1,421	3.70	3.51	3.89
2008	38,330	945	2.47	2.31	2.63	1,273	3.32	3.14	3.50
2009	38,960	1022	2.62	2.46	2.78	1,368	3.51	3.33	3.69
2010	39,573	904	2.28	2.13	2.43	1,459	3.69	3.5	3.88
Total	367,155	7884	2.15	2.10	2.20	12,836	3.50	3.44	3.56

^#^A mother may be admitted to hospital for psychiatric disorders more than one time.

CI: confidence interval.

Table 1 shows an increased trend in hospital admissions for principal diagnosis of psychiatric disorders postpartum over the 10 years between 2001 and 2010 (p < 0.05). The trend was more significant in 2005 and was sustained in subsequent years (p < 0.05). Table 1 also shows that the hospital admission rate for all diagnoses of psychiatric disorders postpartum increased from 2002, peaked in 2005 and then gradually declined in the following years until 2008 (Figure 1) (p < 0.05).

Table 2 shows the OR of hospital admissions rates between 2002 and 2010 compared with the rate in 2001. Similar to the trend in the rate of hospital admissions (Figure 1), hospital admissions for both principal and all diagnoses increased significantly (Table 2) (p < 0.01) compared to 2001. Hospital admissions for principal diagnosis increased more significantly from 2005 (Table 2).

**Figure 2:**
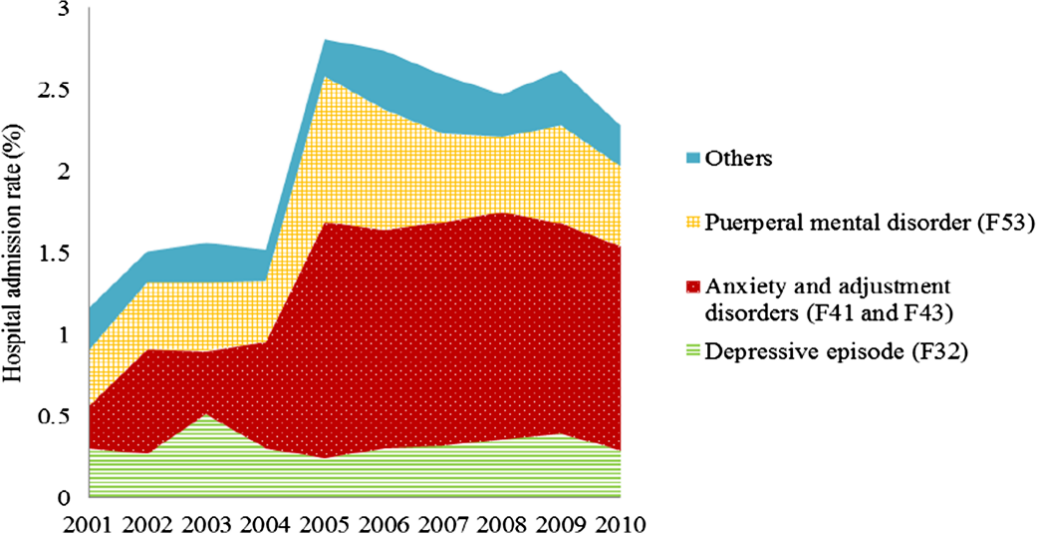
**Hospital admissions for principal diagnosis of psychiatric disorders in primiparous mothers, 2001–2010, New South Wales, Australia.** A mother may be admitted to hospital for psychiatric disorders more than once.

Figure 2 shows the hospital admission rates (in area) by principal diagnosis over the 10-year period. There were significant increases in puerperal mental disorders, anxiety disorders and adjustment disorders. Hospital admission rates for adjustment disorders and anxiety disorders increased at a faster rate than for unipolar depression from 2005 (Figure 2). The increase in overall hospital admissions for principal diagnosis was mainly attributed to anxiety and adjustment disorders. Puerperal mental disorders also contributed to the increase, but not as significantly as anxiety and adjustment disorders.

**Table 2:** **Odds ratio for hospital admissions for psychiatric disorders in primiparous mothers, 2001–2010, New South Wales, Australia**

Year	Primiparous mothers (n)	Principal diagnosis ^#^			All diagnoses ^#^		
		OR	95% CI		OR	95% CI	
2001	34,816	1					
2002	34,463	1.60	1.37	1.86	1.25	1.13	1.40
2003	35,091	1.31	1.12	1.53	1.55	1.40	1.72
2004	34,877	1.60	1.38	1.86	1.89	1.71	2.08
2005	36,157	3.08	2.69	3.54	2.07	1.88	2.28
2006	36,509	2.76	2.40	3.17	1.71	1.55	1.89
2007	38,379	2.56	2.23	2.94	1.59	1.43	1.75
2008	38,330	2.60	2.26	2.98	1.52	1.37	1.68
2009	38,960	2.49	2.17	2.86	1.51	1.37	1.67
2010	39,573	2.34	2.04	2.69	1.72	1.56	1.90

^#^For this analysis, dependent variable was mothers who admitted to hospital for psychiatric disorders in the first year after birth (0 = no, 1 = yes). Mothers who were admitted to hospital more than once in the first year after birth were only counted once.

OR: odds ratio; CI: confidence interval.

For principal diagnosis of puerperal mental disorders, the hospital admissions in 2005 were significantly higher than for other years (p < 0.05). For principal diagnoses of anxiety and adjustment disorders, the hospital admission rates were higher from 2002 than for 2001 (p < 0.05).

**Figure 3:**
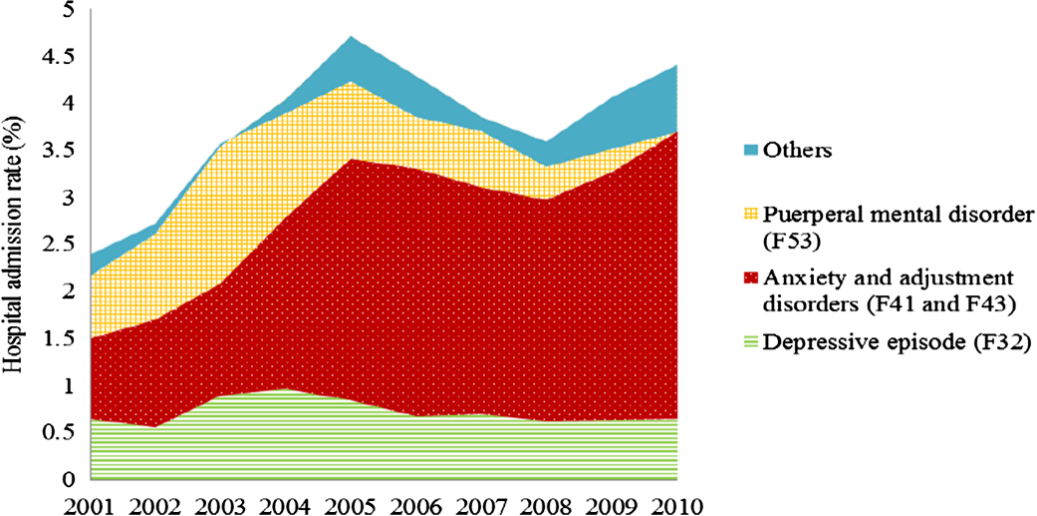
**Hospital admissions for all diagnoses of psychiatric disorders in primiparous mothers, 2001–2010, New South Wales, Australia.** Women with more than one diagnosis in one admission would be counted more than once.

Figure 3 shows the trend in hospital admission rates by all diagnoses over years. For all diagnoses of puerperal mental disorders, the rates of hospital admissions in 2002–2006 were significantly higher than for 2001 (p < 0.05). The hospital admission rates in 2007–2010 were not significantly different from 2001 (p > 0.05). Similar to principal diagnosis, the hospital admission rates for all diagnoses of anxiety and adjustment disorders since 2002 were significantly higher than for 2001 (p < 0.05) (Figure 3). Similar to principal diagnosis, the increase in overall hospital admissions for all diagnoses was mainly attributed to puerperal mental disorders, and anxiety and adjustment disorders.

## Discussion

This study shows an increased trend in the hospital admission rates for both principal and all diagnoses of psychiatric disorders postpartum over a 10-year period between 2001 and 2010, particularly since 2005. This increase in psychiatric hospital admissions postpartum has parallelled the introduction of a number of perinatal mental health initiatives in Australia, and may be associated with improved access to health services for mothers with mental illness [14, 15]. The beyondblue National Action Plan for Perinatal Mental Health recommended training for primary health care professionals to provide effective support to pregnant women and optimise access to appropriate services for their mental health problems. [16] The Better Access to Psychiatrists, Psychologists and General Practitioners through the Medicare Benefits Schedule (Better Access) initiative was introduced by the Australian Government Department of Health and Ageing in November 2006 [17]. It has contributed to an increase in treatment rates for people with mental illness from 35% in 2007 to 46% in 2010 [17]. The National Postnatal Depression Program (2001–2005) increased awareness of perinatal mental illness in health professionals and perinatal women through screening and education [18]. The National Perinatal Depression Initiative established routine depression screening and improved access to mental health services for perinatal women in Australia [19].

This study also showed that the increase in hospital admissions was mainly attributed to anxiety and adjustment disorders (Figures 2 and 3). This is consistent with the interventions resulting from the initiatives and programs mentioned above which mainly focused on anxiety and depression [14, 17, 18].

The changes in service delivery arrangements, such as a shift from psychiatric hospitals to general hospitals, may also contribute to the increase in the hospital admission rate. The aim of the 1993–2008 National Mental Health Strategy was to reduce the size of stand-alone psychiatric hospitals and develop more inpatient services within general hospitals [20, 21]. The study data do not include information on the change of hospital type, so the association between admissions and the initiatives cannot be described in detail at this stage.

In addition to the initiatives and programs, other factors such as maternal age may also explain the increase in mental illness diagnoses for mothers after birth [14]. In NSW, the number of mothers 35 years of age or over giving birth increased from 15,250 in 2001 to 22,991 in 2010, an increase from 18.1% to 24.2% of all pregnancies [22, 23]. One of our previous studies showed that mothers who gave birth at an older age were more likely to be admitted to hospital postpartum for mental illness [24]. The study also showed that having an infant admitted to a neonatal intensive care unit and maternal smoking were both associated with an increased risk of postpartum psychiatric illness [24]. The extent that these factors are associated with the increased trend over time in hospital admissions for mothers’ mental illnesses needs to be investigated further.

Hospital admissions where mental health diagnosis is a non-principal diagnoses should also be considered [25]. This study showed that non-principal diagnoses accounted for 38.58% (4,952/12,836) of all hospital admissions in the study period. Examination of non-principal diagnoses did not suggest difference in severity from principal diagnosis. For example, urgent medical conditions such as postpartum haemorrhage were more likely to be the principal diagnosis. In addition, the average length of stay for non-psychiatric principle diagnoses comorbid with MBD was significantly longer than general diagnoses [26]. For MBD as non-principle diagnoses, the data for this study did not distinguish between those that complicated the treatment and/or extended the length of stay and those well controlled. Further studies should investigate the impact of MBD as non-principle diagnoses on hospital admissions.

This study was limited to hospital admission rates. The use of outpatient services was not included. These data do not include the variable which shows the introduction of relevant policy changes in NSW. It is difficult to find the direct reason for the significant increase of mothers’ hospital admissions for psychiatric disorders during the 10-year period. For the hospital admissions with psychiatric disorders as a non-principal diagnosis, the reason for the admission may be non-psychiatric diagnosis. The impact of mental health when the principal diagnosis is non-principal diagnosis requires further examination.

## Conclusion

This study shows that the hospital admission for mothers with mental illness after birth in NSW (Australia) has significantly increased in the last decade. Possible reasons for this change need to be studied further. The state-wide policy initiatives and associated educational initiatives and public awareness campaigns have likely increased awareness among both health care providers and the public and thus no doubt play an important role in the increase in admissions observed.

## Abbreviations

AIHW: Australian Institute of Health and Welfare
APDC: Admitted Patients Data Collection
CHeReL: Centre for Health Record Linkage
CI: Confidence interval
MBD: Mental and behavioural disorders
MDC: Midwives Data Collection
NSW: New South Wales
OR: Odds ratio.
